# Identification and high-throughput genotyping of single nucleotide polymorphism markers in a non-model conifer (*Abies nordmanniana* (Steven) Spach)

**DOI:** 10.1038/s41598-023-49462-x

**Published:** 2023-12-15

**Authors:** Kedra Ousmael, Ross W. Whetten, Jing Xu, Ulrik B. Nielsen, Kurt Lamour, Ole K. Hansen

**Affiliations:** 1 Department of Geosciences and Natural Resource Management, University of Copenhagen, Rolighedsvej 23, 1958 Frederiksberg C, Denmark; 2 Department of Forestry and Environmental Resources, North Carolina State University, Raleigh, NC 27606 USA; 3 Department of Entomology and Plant Pathology, University of Tennessee, Knoxville, TN USA

**Keywords:** Biotechnology, Genetics, Plant sciences

## Abstract

Single nucleotide polymorphism (SNP) markers are powerful tools for investigating population structures, linkage analysis, and genome-wide association studies, as well as for breeding and population management. The availability of SNP markers has been limited to the most commercially important timber species, primarily due to the cost of genome sequencing required for SNP discovery. In this study, a combination of reference-based and reference-free approaches were used to identify SNPs in Nordmann fir (*Abies nordmanniana*), a species previously lacking genomic sequence information. Using a combination of a genome assembly of the closely related Silver fir (*Abies alba*) species and a de novo assembly of low-copy regions of the Nordmann fir genome, we identified a high density of reliable SNPs. Reference-based approaches identified two million SNPs in common between the Silver fir genome and low-copy regions of Nordmann fir. A combination of one reference-free and two reference-based approaches identified 250 shared SNPs. A subset of 200 SNPs were used to genotype 342 individuals and thereby tested and validated in the context of identity analysis and/or clone identification. The tested SNPs successfully identified all ramets per clone and five mislabeled individuals via identity and genomic relatedness analysis. The identified SNPs will be used in ad hoc breeding of Nordmann fir in Denmark.

## Introduction

The world’s productive forests are facing significant challenges from climate change, the green transition, and a growing global population that is driving up demand for wood products. Forest tree breeding, assisted migration, and other forms of genetic management seem to be evident and realistic solutions to address these challenges, both in terms of the necessary adaptation to new environments and of increasing wood production. Traditional long-term forest tree breeding with field trials is likely unrealistic, except for the most important timber species^1^. Forest tree breeding could, however, be accomplished via ad hoc breeding, using pedigree reconstruction with DNA markers in production stands^2^. For the assisted migration of forest tree species and/or seed sources, genetic monitoring and management is imperative. Working on many species in many forest stands and genotyping many thousands of individuals brings with it the need to develop cost-effective, high-throughput DNA markers. The present study explores methodologies that could be applied to generate DNA markers in a conifer species. This process is particularly challenging, because of the relative scarcity of unique, non-repetitive DNA sequences in conifers^3^, which is a prerequisite for informative DNA-markers with mendelian inheritance that can be used in e.g. pedigree reconstruction.

Single nucleotide polymorphisms (SNPs) are the most frequent form of variation in genomic DNA, where different sequence alternatives (alleles) occur at a single base level^4^. Besides being the most frequent form of variation in eukaryotic genomes, SNPs are primarily biallelic, ubiquitous, and amenable to high-throughput automation^5,6^. Single nucleotide polymorphisms have been successfully applied in genetic marker-assisted breeding in numerous economically important species with time and cost savings; this is especially important in woody perennials^7^. Although the focus has hitherto been on microsatellites, SNPs are replacing this marker type in kinship analysis and pedigree reconstruction because of the advantages wrought by high-throughput genotyping.

Single nucleotide polymorphism identification typically involves mapping sequences onto a reference genome followed by a variant calling step to identify the SNP positions. However, in the case of non-model species, especially conifers with large and complex genomes, the availability of even a single reference genome remains limited to very few species (i.e., *Picea abies*:^8^; *Pinus taeda*:^9^; *Pinus lambertiana*:^10^; *Pseudotsuga menziesii*:^11^; *Abies alba*:^12^). Owing to their large size (≥ 18–20 Gb) and repetitive character, none of these reference genomes are complete. The recently published genome of the Chinese pine (*Pinus tabuliformis*;^13^, coast redwood (*Sequoia sempervirens*;^14^), giant redwood (*Sequoiadendron giganteum*;^15^) and Japanese cedar (*Cryptomeria japonica*;^16^) are the only chromosome-level assemblies. Some of the alternatives used in the absence of reference genome includes, RNA-seq, Genotyping-by-Sequencing^17^ and other reduced representation based techniques, such as RADseq^18^ and DArTseq ^19^. RNA-seq can provide information about gene expression levels while at the same time enabling SNP detection in the expressed region. However, RNA-seq only offers a modest amount of coverage (expressed region). In addition, it could result in detection of variants that are introduced by RNA editing, hence not present at the DNA level. Moreover, a highly variable nature of gene expression could make SNP detection a challenging task^20^. On the other hand, genetic analysis has been made possible in a substantial number of non-model plants thanks to Genotyping-by-Sequencing and similar technologies (such as DArTseq and RADseq). They can, however, be susceptible to batch effects, and between-sample differences frequently produce a considerable quantity of missing data, which has an impact on the consistency and quality of results across samples and studies. Furthermore, even though they can be economical when genotyping a modest number of samples, they might be prohibitively expensive when working with big populations.

Though not widely applied, a reference-free SNP identification approach that detects variants directly from primary sequences has recently been developed for use in the absence of high-quality reference genomes^21–23^. Ebwt2InDel^23^ is one of the reference free SNP calling approaches. It uses positional clustering theory^24^ to detect SNPs using extended Burrows-Wheeler-Transform of a collection of reads. The basic principle in the positional clustering theory is that the extended Burrows-Wheeler Transform (eBWT) of a collection of reads tend to cluster together bases that span the same genomic location. These clusters are identified using the *Longest Common Prefix Array* and the *Suffix Array* of the dataset. This clustering enables locating and analyzing this genome positions to identify SNPs^23,24^.

Conifers have worldwide economic importance because they are amenable to large-scale wood production, rapid growth, and relative ease of both paper and solid wood processing^25^. In addition, owing to their attractive foliage, some coniferous species are in high demand for their ornamental value, while others are required for special purposes such as Christmas trees. Nordmann fir (*Abies nordmanniana* (Steven) Spach) is one of the most prominent Christmas tree species, with over 40 million trees sold annually in Europe alone^26^.

Denmark is the only country with a breeding program for Nordmann fir Christmas trees^27^, and references therein]. The program, which began in 1992, has relied primarily on seed harvesting of selected plus (i.e., best performing) trees and their use in traditional half-sib field trials. In parallel, clonal seed orchards (CSOs), which are grafted with scions from the plus trees, can be genetically thinned when the half-sib trials had been evaluated after a Christmas tree rotation. Nordmann fir reaches reproductive maturity at the late age of 30–35 years. Therefore, many of the plus trees have been chosen at an age beyond Christmas tree size, when the focus has been on health and needle appearance. However, in a specific part of the breeding program—the so-called Ambrolauri gene pool—plus trees have been selected in Christmas tree stands 12–14 years from seed, which is the normal age for felling. Scions from the plus trees have been grafted in CSOs, but no seed for half-sib trials has been available (because they are so young). The resulting CSOs belonging to the Ambrolauri gene pool delivered their first seed crops in 2009, and many commercial Christmas tree stands based on their seed production have since been established. To be able to carry out a genetic thinning of the first generation CSOs in the Ambrolauri gene pool and select the best plus trees for the second generation, an ad hoc breeding approach was undertaken. In this breeding activity, parentage analysis had to be conducted for thousands of trees, using DNA-markers, and it was therefore decided to develop a panel of SNP markers.

Nordmann fir has a rather limited natural distributional range in the Caucasian region. This includes Georgia, the southern part of Russia, and the northeastern parts of Turkey^28^. The species tends to be used primarily as a Christmas tree, so it has high regional economic importance (e.g., in Denmark and northern Germany). There is hardly any genomic information thus far (e.g., no reference genome).

The present study aimed to explore a combination of reference-based (using the closely related Silver fir genome) and reference-free approaches to develop a set of reliable SNPs in Nordmann fir, thereby enabling ad hoc breeding. The SNPs thus developed will help significantly in the ongoing breeding program of the species.

## Methods

### Plant material and DNA extraction

Two commercial seed lots from the CSOs FP. 259 and FP. 266, respectively, obtained from Nature agency in Denmark, were used as starting material. These two CSOs represent two different gene pools in the first generation of the Danish Christmas tree breeding program^29^, namely:

A. The Borshomi gene pool—where FP.259 currently consists of 68 clones from the approved Danish seed stands F.526 (17 clones) and F.527 (43 clones; 1st generation seed imported directly from the Borshomi area in Georgia), as well as from the Lilleheden source (8 clones), which was the second generation from seed directly imported from the Borshomi area.
B. The Ambrolauri gene pool—where FP.266 currently consists of 143 clones that have been selected from the approved Danish seed stands F.808 Ny Saltbjerg and F.824 Tveden, both of which originate from the Ambrolauri area in Georgia.

Two seeds from each of the two seed lots were soaked in de-ionized water for 24 h before the embryos and the seed skin was removed. DNA was extracted from the megagametophytes using the DNeasy Plant Mini Kit from QIAGEN (Germany). Low-coverage whole genome sequencing was carried out using a NovaSEQ 6000 running a 2 × 150 bp configuration at Admera Health LLC. Library preparation included shearing to roughly 300 bp and dual-index, PCR-free library construction and quantification using KAPA kits, according to the manufacturer’s instructions. This resulted in approximately 300 million pairs of 150 bp for each of the four samples (around 2.4 billion sequences in total). A total of 342 individuals representing 140 clones originating from the Ambrolauri gene pool was used to validate the identified SNPs.

### Data preprocessing and assembly

FastQC^30^ was used to obtain an overview of the quality status of the raw sequencing reads per sample. Individual FastQC reports were then summarized in an overall quality status report by MultiQC^31^. All reads were trimmed for adaptor sequences, G-homopolymer and poor-quality bases (< 10 quality score) using FastP^32^.

Up to 75% of conifer genomes is made up of highly repetitive DNA sequences^33^. These often pose a challenge during assembly and marker identification. Given the difficulty of resolving repetitive regions with low-coverage sequencing, we used reads originating from low-copy regions of the genome for de novo assembly. As a first step in repeat detection and de novo assembly, KMC v3^34^ was used to count k-mers, a procedure that determines all unique substrings of length k in the sequencing reads. The k-mer database was converted into a histogram text file using the *kmc_tools transform* program^34^. The *kmc_tools filter* program was then used to filter the input reads and recover putative low copy reads. The filtered low copy reads were then correctly paired into read1 and read2 sequences and unpaired reads were written to a separate singletons file using the *repair.sh* tool from the BBTools Suite^35^.

**Figure 1:**
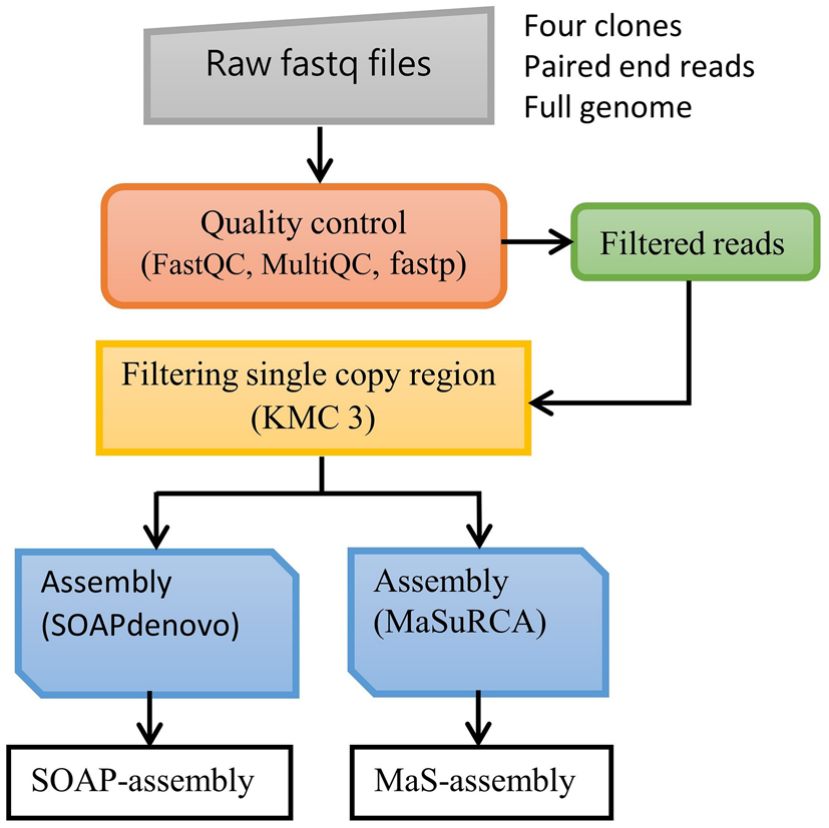
Schematic workflow showing the de novo short read assembly approaches used with respective quality control, filtering and assembly tools indicated in brackets.

Two de novo assembly tools, namely MaSuRCA^36^ and SOAPdenovo2^37^ version r240, were used to assemble the filtered reads into contigs (Fig. 1). In the preparation of the config file for MaSuRCA, the k-mer size was left to auto so the program could select the optimal one, and the k-mer size of 99 was selected for the graph reconstruction. The same k-mer size of 99 was also used to assemble reads using SOAPdenovo2.

### Mapping of short-read sequences

For simplicity, we compared the two de novo assemblies from MaSuRCA and SOAPdenovo2 (hereafter denoted as MaS-assembly and SOAP-assembly) based on simple criteria, that is, assembly statistics and mapping quality (length and percent identity of the alignment) to the Silver fir genome. Consequently, the MaS-assembly was selected.

In addition to the MaS-assembly, a draft genome from a closely related species, Silver fir (*Abies alba*), version Abal.1_1.fa, was used as a reference^12^. The four haploid individual read files were mapped to the two references using BWA^38^. Samtools^39^ *flagstat* was used to generate descriptive statistics of the alignment.

Mapping the entire haploid individual reads to the MaS-assembly resulted in unrealistic mapping and SNP frequency over estimation. Up to 53.2% of the reads from the individual haploid samples were aligned to the low-copy assembly that constitute less than 3% of the genome in size. This indicates that BWA found the best possible alignment for each read or read pair in the low-copy assembly, even though the read may not have originated from that particular region of the genome when the sequencing library was constructed. The expected depth of coverage of each of the haploid samples is 5x, considering the Nordmann fir genome size. Meanwhile, the average depth based on mapping to the Silver fir genome is 7x. This implies that each contig in the low-copy assembly would align to about 7x coverage of reads, with some variation due to random sampling. However, the depth of coverage value in the BAM files was up to ten times higher (average = 39), with more than 90% and 60% of the regions having a depth higher than the  expected (based on mapping to the Silver fir genome) and higher than twice the expected, respectively.

To overcome this, we carried out the following steps:

- The MaS-assembly was mapped to the Silver fir genome to produce BAM files;
- BEDtools intersect^40^ was used to produce a list of read identifiers for each haploid sample aligned to the subset of the Silver fir genome where contigs from the low-copy assembly align;
- The seqtk toolkit^41^ was used to subset those reads from the original haploid samples;
- The subsets of reads from each haploid sample were mapped to the low-copy assembly.

Another solution to the excessive depth issue is to exclude regions with unrealistically high depth, either from the BAM files or later at variant filtering stage. However, given the small size of the de novo assembly and a highly repetitive nature of the Nordmann fir genome, reads originating from similar regions (e.g., paralogous sequences) could map on top of each other and still pass a filtering threshold of maximum depth ($d+4\sqrt{d}$,^42^ for instance, where *d* is the average depth). 

### SNP calling

**Figure 2:**
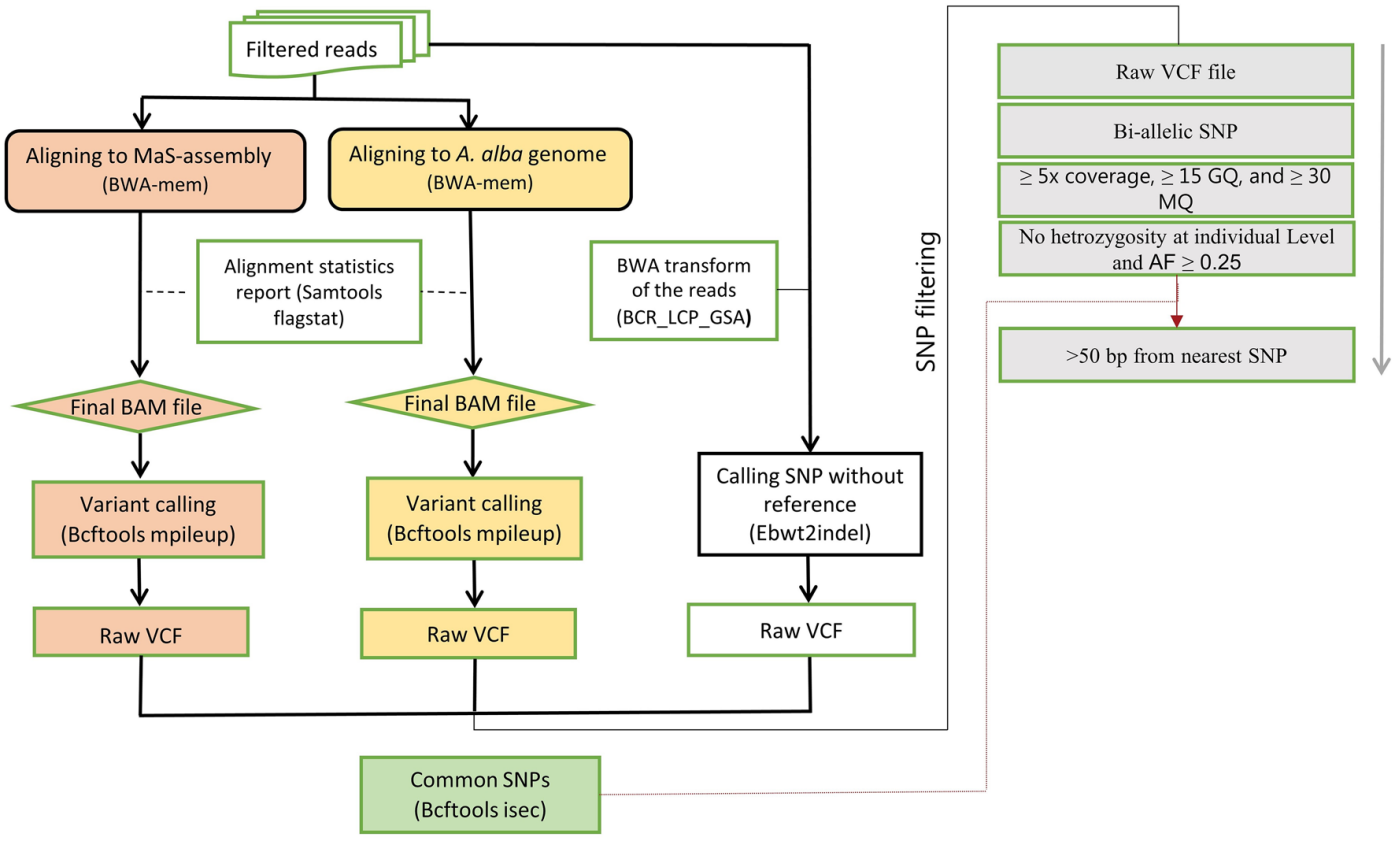
Schematic workflow showing the major steps in the three different SNP calling approaches used (two reference-based and one reference-free). The first two columns colored peach, and yellow indicate reference-based approaches using MaS-assembly, and *A. alba* (Silver fir) references, respectively. The third column indicates a reference free approach using ebwt2InDel. The right most column illustrates the SNP filtering pipeline used.

Both reference-based (calling SNPs from alignment) and reference-free approaches were used to call SNPs (Fig. 2). Bcftools *mpileup*^43^ was used to call SNPs from the alignments. Meanwhile, ebwt2InDel^23^ was used as a reference-free SNP calling approach. Ebwt2InDel discovers SNPs/indels inside one set (heterozygous sites) or between sets of reads (fasta/fastq) without aligning them to a reference genome.

To call SNPs without a reference using ebwt2InDel, the BCR_LCP_GSA program was first used to create a Burrows Wheeler Transform (BWT) of the reads, from which ebwt2InDel calls SNPs. To enable comparison with SNPs identified with the reference based approaches, the ebwt2InDel calls were converted to a Variant Call Format (VCF) using the Silver fir genome and MaS-assembly.

Raw VCF‐files across the four haploid genomes were filtered through several steps. First, only the biallelic SNPs were retained. SNPs with < 5x coverage, < 15 genotype quality, and < 30 mapping quality were excluded. Because haploid megagametophyte tissue was used as a source of DNA, all loci showing heterozygosity at the individual level were filtered out. In addition, because of the haploid sequence and only four individuals used for SNP identification, any polymorphic SNP has an alternative allele frequency (AF) of at least 0.25, and as a result, only SNPs with AF ≥ 0.25 were kept at this filtering stage.

The number of shared SNPs detected among each of the reference-based and reference-free approaches was identified using the Bcftools *isec.* This allowed us to select SNPs identified by different SNP calling methods. However, because two different references were used in the reference-based approaches, it was impossible to carry out direct intersection analysis and identify which SNPs from the whole genome alignment were present in the assembly generated by MaSuRCA. Thus, an indirect approach was used. The alignment of the MaS-assembly to the Silver fir genome was used to obtain the bed file with the exact coordinates (scaffold ID, alignment start, and alignment end) where the assembly was aligned. The coordinates were used to obtain a subset of variants that were identified within the coordinates that were polymorphic among the four samples. Figure 2 shows an overview of the SNP-calling pipeline.

### SNP genotyping primer design and targeted sequencing

Primer3 v2.5.0^44^ was used to design targeted amplicon sequencing primers (200 pairs) for a selected subset of the identified SNPs. Out of the 200 tested SNPs, 50 were detected by all methods, while 50 were shared between Silver-based and reference-free methods. Additionally, 50 SNPs overlapped between MaS-based and Silver-based methods, and another 50 were common between MaS-based and reference-free methods. The selected SNPs are located on different contigs. We used needles as a source of DNA for amplicon sequencing. Targeted amplicon sequencing of 342 Nordmann fir individuals from FP.266 was accomplished using the Hi-Plex approach^45^ at Floodlight Genomics LLC, and the resulting amplicons (80–100 bp) were sequenced on the Illumina NovaSEQ 6000 according to the manufacturer’s instructions at Admera Health LLC, Plainfield, NJ, USA. The 342 individuals comprised 88 clones with three ramets per clone, 26 clones with two ramets, and 26 where the ortet (the original plant from which a clone is started) was represented by a single ramet only. The amplicon sequences were first processed using Fastp^32^ to remove adapter sequences and reads with a Q score below 20. The filtered reads were mapped to references—Silver fir for the 150 SNPs at an intersection of Silver-based SNPs and the other two methods: MaS-assembly for the 50 SNPs common between MaS-based and ebwt2InDel SNPs. We used ANGSD v. 0.940^46^ for genotype calling. The filtering criteria used in ANGSD include discarding bad reads (flag ≥ 256); discarding reads that did not map uniquely, and keeping only those with mapQ > 20. Moreover, only truly variable SNPs (*p* values less than 0.001) were kept. Posterior genotype probability calculation was also enabled to keep sites with > 0.95 posterior genotype probability. The selected SNPs were tested in the context of identity analysis.

### SNP validation

Reproducibility of the SNPs was evaluated by determining whether any two or three ramets had identical genotypes across all the tested SNPs. For this purpose, we calculated the percentage of mismatches between the 83 ramet trios with confirmed identity and an equal number (83) of randomly assigned unrelated trios. In addition, to evaluate the change in number of mismatches between trios and duos, we compared an equal number of pairs (83 pairs randomly selected from the 83 ramet trios). To make a group of unrelated pairs, we randomly assigned 83 pairs and trios of unrelated individuals across clonal groups. We compared the mismatch profile of the ramets with that of unrelated individuals. Secondly, we conducted identity analysis in CERVUS v. 3.0.7 software^47^. Finally, we computed a genomic relatedness matrix^48^ for all tested clones.

### Plant guidelines

The collection and performance of experimental research on Nordmann fir samples in this study complied with the national guidelines of Denmark.

## Results

### Quality status of the sequencing reads

The number of paired-end reads of the four individuals before quality filtering ranged from 556.8–602.1 million (M) reads. The number of reads remained almost the same after filtering (556.3–601.6). Overall, 99.9% of reads passed quality filtering. The minimum mean quality value across each base position in the read across the four clones before filtering was 34.9, while the maximum was 36.6.

### Sequencing coverage

The k-mer analysis did not show a second peak corresponding to k-mers that occur once in the genome and resolves errors from single-copy k-mers. To explore this, different k-mer lengths were tried in different k-mer counting programs, including KMC v3, Jellyfish, and *kmercountexact.sh* from BBmap. However, the histograms from all these programs showed a steady and consistent decrease in k-mer abundance distribution, without any sign of the second peak. To ensure that this issue was not the result of contamination of the sequencing reads with other species, we carefully analyzed the quality of the reads. We also mapped the filtered reads to the Silver fir genome and analyzed the alignment for mapping quality and percent identity. This enabled us to rule out contamination as the reason for the absent peak. This might be an indication that the rate of variation is very high in the genome.

The sequencing coverage was determined using data from the Kew database^49^ regarding Nordmann fir DNA content. This data was combined with information on the average number of reads per sample and the average read length. Specifically, the total sequence per individual was calculated by multiplying the average number of reads per individual (592.9 million reads) by the average read length (149 bases), resulting in 88 gigabases (Gb). The expected coverage was then obtained by dividing this total sequence per individual by the expected haploid DNA content of 17.3 Gb, yielding an approximate coverage of 5x.

### De novo assembly and mapping

The filtered reads of the four individuals were merged into a single file to obtain around 20x coverage. To obtain a collection of reads to be assembled, the merged read files were filtered using the *kmc_tools filter* program to keep only reads that contained 90% to 100% k-mers with coverage between 10x (one-half the expected coverage) and 40x (twice the expected coverage).

Assembly of the filtered low copy reads using MaSuRCA and SOAPdenovo2 resulted in total assemblies of 399 Mb and 676 Mb, respectively (Table 1).

**Table 1:** Assembly statistics for the two de novo assemblies (Mb = Megabase).

Assembler	Sum contig length	Mean size of contigs	N50
MaSuRCA	399 Mb	569 bp	593 bp
SOAPdenovo2	676 Mb	469 bp	464 bp

The percentage of mapped reads varied between an average of 97.7% in reads mapped to the MaS-assembly and an average of 99.7% in reads mapped to the Silver fir draft genome (Table 2). It should be noted that only a subset of reads originating from the 1−2x copy regions were mapped to the MaS-assembly.

**Table 2:** Alignment summary statistics (Mapped = proportion of reads aligned to a reference; Properly paired = both mates of a read pair map to the same contig, oriented towards each other, and with a reasonable insert size; With itself and mate mapped = total mapped reads including those that are not properly paired; Singletons = only one mate in a pair is mapped).

Alignment	Reads passed QC (M)	Mapped (%)	Properly paired (%)	With itself and mate mapped (M)	Singletons (%)
Silver_9	611.9	99.8	82.5	599.3	0.1
Silver_12	603.1	99.7	82.7	590.3	0.1
Silver_13	631.5	99.7	82.8	618.2	0.1
Silver_15	566	99.8	83.7	554.7	0.1
SOAP_9	37.4	97.7	80.2	29.0	0.7
SOAP_12	36.7	97.7	80.3	28.5	0.7
SOAP_13	38.9	97.7	80.2	30.2	0.8
SOAP_15	35.0	97.8	80.3	27.2	0.7
MaS_9	20.5	97.0	88.9	16.5	0.9
MaS_12	20.2	97.0	88.9	16.3	0.9
MaS_13	21.4	96.9	88.8	17.2	0.9
MaS_15	19.2	97.0	89.0	15.5	0.9

The alignment names include the reference used followed by the individual ID.

### Proportion of SNPs

The number of raw SNPs detected from the reads mapped to the MaS-assembly and the Silver fir reference was 6.8 M and 464 M, respectively (Table 3). After filtering, the SNP numbers decreased to 2.07 M and 98.1 M in reads mapped to MaS-assembly, and Silver fir references, respectively. The reference-free approach using ebwt2InDel detected 8.1 M SNPs.

**Table 3:** Number of SNPs identified in different approaches.

Approach	SNP caller	Reference	Raw SNPs (M)	Filtered SNPs (M)	> 50 bp apart (M)
Reference-based	Bcftools-mpileup	MaS-assembly	6.8	2.07	1.3
		*A.alba*	464	98.1	49.5
Reference-free	ebwt2InDel	–	8.1	8.1	6.6

### Shared SNPs

**Figure 3:**
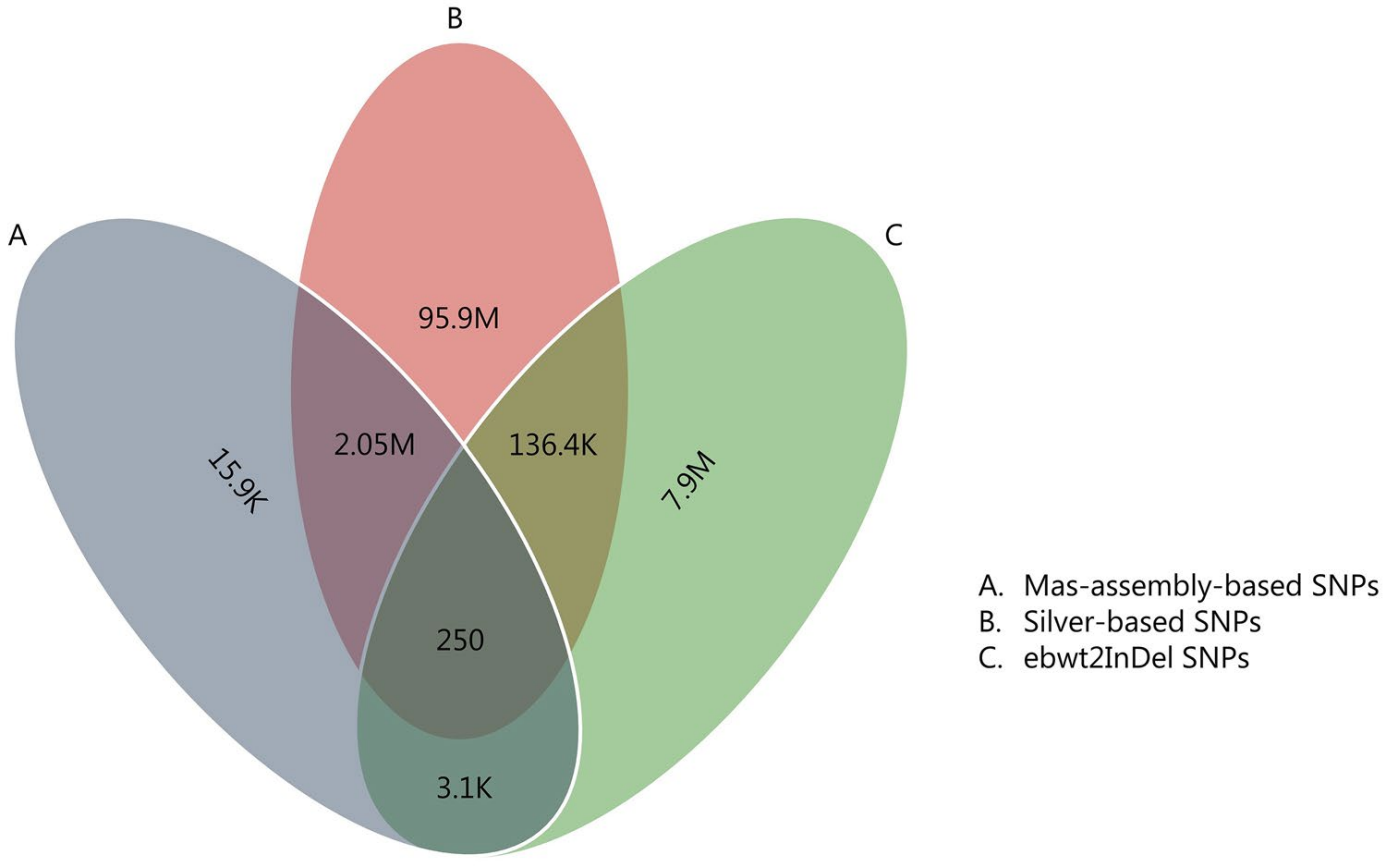
Venn diagram showing the proportion of shared SNPs among the different SNP identification approaches.

The two reference-based approaches detected 2.05 million SNPs in common (Fig. 3). Of these, only 250 were found among the SNPs detected by the reference-free (ebwt2InDel) approach. However, Silver-based SNP calling and the reference-free approach detected 136.7 K SNPs in common, while MaS-based and reference-free approach detected 3.4 K SNPs in common.

**Figure 4:**
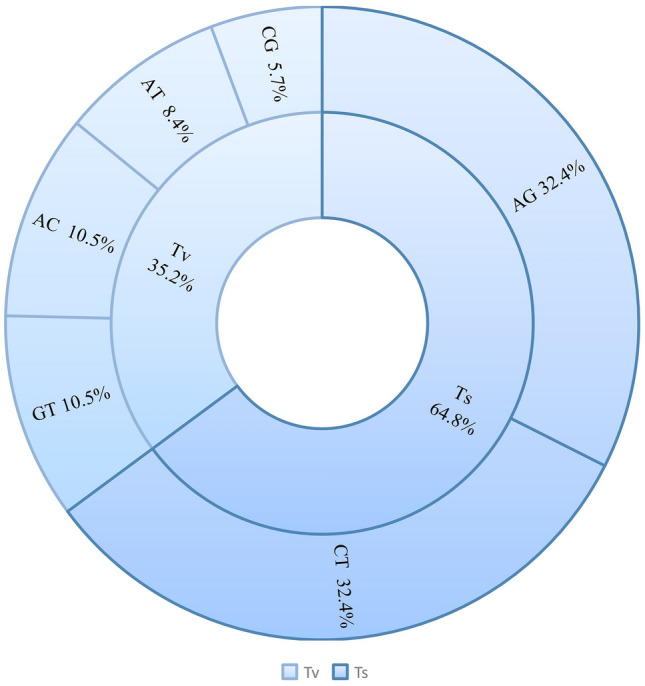
Proportion of different types of nucleotide substitution among the 2 million SNPs from the Silver fir alignment also found in the MaS-assembly.

The ratio of transition (Ts) to transversion (Tv) among the shared SNPs is 1.84 (Fig. 4). Among the four transversion substitutions, the A–C and T–G were more common than the A–T and C–G substitutions. The C–G substitution was the least common.

### SNP genotyping of Nordmann fir clones

As a SNP validation step, primers were designed for a subset of 200 SNPs (Supplementary material) identified in common among the three approaches (including the reference-free one). The designed primers were used for targeted amplicon sequencing and subsequent genotyping of 140 clones from the Ambrolauri gene pool (= selected trees grafted in the CSO FP.266). Out of the 200 tested primer pairs, 197 resulted in amplicons with an average minimum coverage depth of 21x, which was used for genotype calling. We obtained 193 SNPs with a *p* value of 0.001 and a posterior genotype probability > 0.95 from genotyping. The final set of genotyped individuals was 342, including 88 clones that had three ramets per clone, 26 clones with two ramets, and 26 where the ortet was only represented by a single ramet. From the 193 genotyped SNP loci, those with ≥ 15% missing data were excluded. In addition, minor allele frequency was used as further filtration criteria, where loci with minor allele frequency ≤ 0.05 were filtered out. Missing data across each sample was also calculated to exclude individuals with missing data in > 20% of the loci. The filtered data had all 342 genotyped individuals representing all 140 clones and 169 SNPs (87.5% of the successfully genotyped SNPs). From the four categories of tested SNPs, 46 of the ones shared by all approaches, 45 of the SNPs shared between MaS-based and ebwt2InDEL, 40 of the SNPs in common between Silver-based and ebwt2InDEL, and 38 of the SNPs shared between MaS-based and Silver-based SNPs constituted the 169 SNPs. Furthermore, 56 SNPs with heterozygosity significantly higher than 0.5 (according to confidence intervals), where 0.5 is the theoretical expected maximum for the population mean heterozygosity in a loci with two alleles, were excluded for the genomic relatedness analysis. Heterozygosity within the remaining 113 SNPs ranged from 0.03 to 0.57 with an average of 0.36.

### Clone analysis

**Figure 5:**
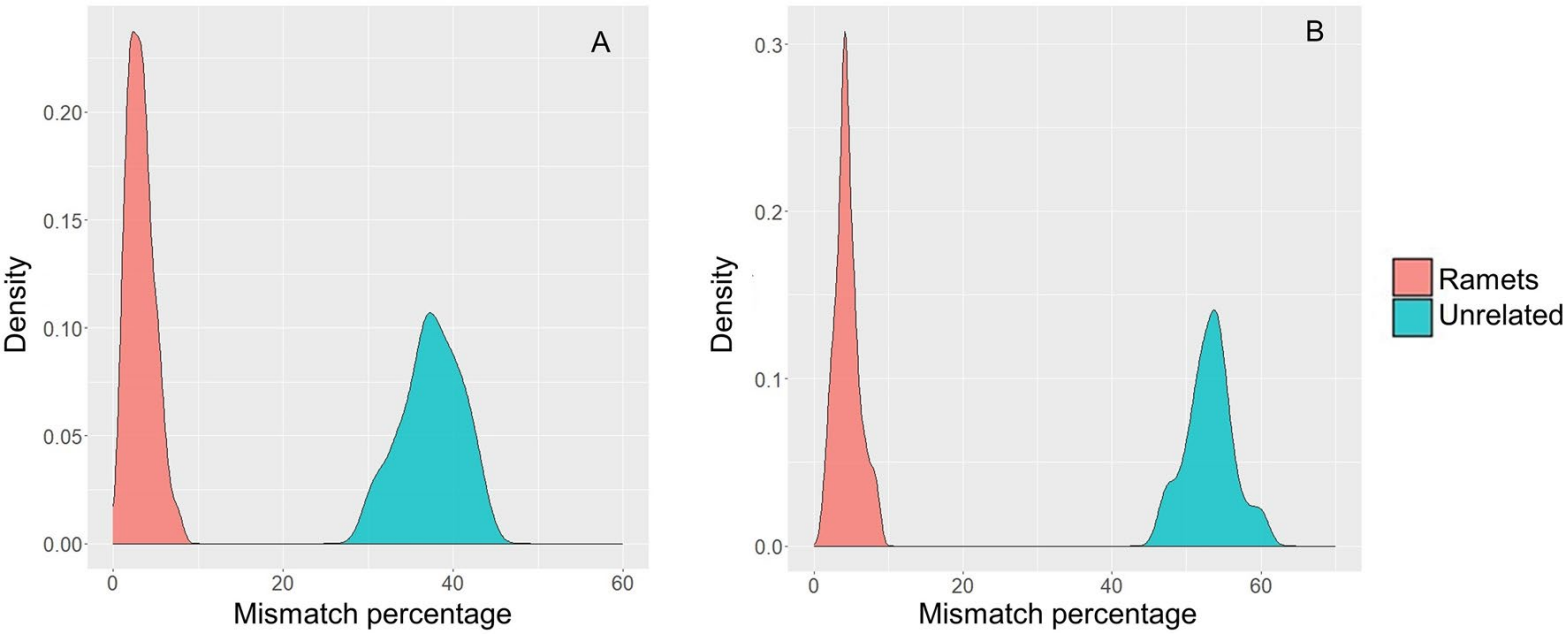
Density plot of mismatch percentages for ramets and unrelated groups, where plot A shows the mismatch percentage for pairs and plot B shows the mismatch percentage for trios.

As a first step in clone analysis, we compared the percentage of mismatches between ramets to the percentage of mismatches between unrelated individuals. As expected, there was a large gap in the percentage of mismatches between ramets and unrelated individuals. The mismatch between ramet trios ranged from 1.0 to 8.8%, while the mismatch between ramet pairs ranged from 0.5 to 7.8% (Fig. 5). The mismatch range was 30.1–43.5% in an unrelated pair group and 46.6–60.6% in an unrelated trio group. The largest gap (37.8%) in the number of mismatches, i.e., the gap between the maximum mismatch percentage for ramets and the minimum mismatch percentage for unrelated individuals, was observed in the trio comparison. The mismatch percentage reported here was calculated after the mislabeled ramets were reassigned to the right clone.

**Figure 6:**
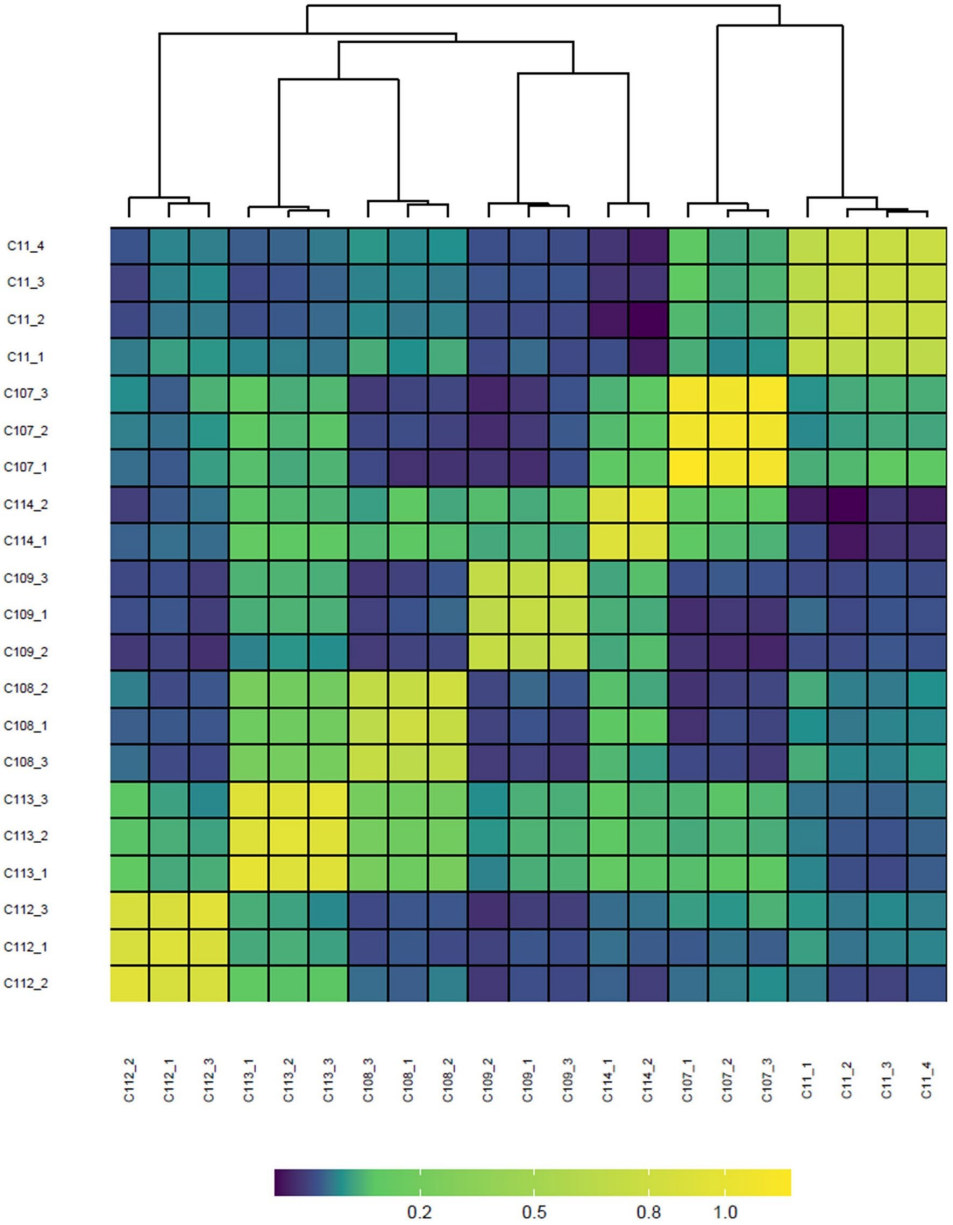
Heatmap of genomic relatedness between the tested clones and their ramets; only part of the heatmap is shown. The genomic relatedness matrix was calculated according to^48^ using 113 SNPs. The small yellow boxes along the diagonal represent ramets of the same clone.

Following a comparison of the mismatch profiles, identity analysis was carried out by allowing a fuzzy match where all ramets were assigned to a clone. The genomic relatedness matrix computed using the 113 selected SNPs confirmed the identity of the ramets per clone (Fig. 6). We were able to reconstruct a similar matrix with just 50 SNPs. In addition, both identity analysis and the genomic relatedness matrix identified 5 mislabeled individuals, four of which were reassigned to another clone and one was unrelated to any other individual. Although the clones were mostly unrelated, some showed a degree of relatedness (*r* =  ~ 0.25) that was close to a half-sib relationship.

## Discussion

We have herein presented our approach for SNP identification in a species without a reference genome. It combined a reference genome from a closely related species with a de novo assembly of low-copy regions to identify reliable SNPs. We also employed a reference-free SNP identification approach. The SNPs identified across the different approaches were tested in the context of identity analysis.

We used haploid megagametophytes as a source of DNA for the full genome sequencing. Haploid tissues are useful for the genomic study of species with large genomes^50^. They can be used as a first step in sequence complexity reduction. Moreover, haploid sequences are easier to assemble than diploid sequences^51^. The content of chloroplast DNA is also very low in the megagametophyte tissue. As a result, the sequencing capacity is not wasted on this DNA type, even when using a standard DNA extraction protocol. We used high-throughput next generation sequencing (NGS) technology to obtain a full genome sequence of four individuals/trees representing two gene pools. From the four sequenced samples, an average of only 2% of the reads mapped to the chloroplast genome of Silver fir. To overcome the challenges associated with assembling a large and complex genome, we pooled together the non-repetitive parts from the four individuals to boost coverage to 20x. We used the pooled non-repetitive parts as an input for assembly to obtain references for SNP identification. Even for Sanger sequencing that produces DNA fragments of up to 1000 base pairs, assemblers often require around 8x coverage of each piece of the genome to compensate for missing and erroneous parts^52^. Next-generation sequencing (Illumina sequencing) appears to require a higher read depth compared with Sanger sequencing^53,54^. This is because assembly results are highly influenced by the amount and distribution of repeats. High repeat content results in non-contiguous assembly because the assembly algorithms are unable to discern the proper assembly of these areas^55^.

The two applied de novo assemblers resulted in assemblies with significant difference in total length, with the SOAP-assembly being 676 Mb and the MaS-assembly being 399 Mb. Despite the smaller overall contig length, the mean contig length and N50 were higher in the MaS-assembly. This may have been because MaSuRCA collapses regions of related sequences into one consensus sequence whereas SOAPdenovo2 keeps them as two separate contigs. Collapsing regions of allelic difference into a single contig is ideal for SNP detection, as it would be impossible to detect SNPs if each allele aligns to a contig that exactly matches its sequence. On the other hand, collapsing related sequences into a single contig would artificially inflate the number of SNPs detected in that region. Comparing different assemblies is, however, a challenging operation that necessitates rigorous analysis and may require inputs from different assemblies to report a good target sequence^56^. However, for the purpose of this study, we selected MaS-assembly due to its better contiguity and percent identity to the *A. alba* genome.

Given the uncertainties associated with using related species as a reference, it is important to ensure the accuracy of identified SNPs. The same uncertainty applies to using assemblies made from low coverage sequencing, as it is difficult to discern true SNPs from sequencing errors. To solve these problems, we used a combination of two different references: the Silver fir genome and a de novo assembly by MaSuRCA, where only low-copy regions of the genome (1–2x) were assembled into contigs to identify SNPs from these regions. The two references detected two million SNPs in common. We believe these SNPs to be more accurate than SNPs identified by individual references, as the probability of the same erroneous SNP being in the two approaches is low.

The number of SNPs identified by the reference-based approaches was high. The 98.1 million SNPs identified from the Silver fir alignment corresponds to one SNP every 176 bases when the haploid genome size of Nordmann fir is taken into account (or 1 every 185 bases when the haploid genome size of Siver fir, 18.16 Gb^12^ is taken into account), indicating high genetic variation. The presence of many SNPs in the Silver fir alignment could be interpreted as a consequence of using a closely related species as a reference. However, only SNPs polymorphic between the four Nordmann fir individuals were kept. Despite the relatively small sizes of the MaS-assembly, which was only 399 Mb in length, a comparable number of SNPs were identified from the de novo assembly. The number of SNPs in the MaS-assembly corresponds to one SNP every 193 bases, likewise indicating the presence of high variation in the genome. While there is no report on the level of genetic polymorphism in Nordmann fir using SNPs, Hrivnák et al.^57^, leveraging microsatellites, reported the presence of high genetic variation in their study where North-Turkish, Georgian and Russian populations of Nordmann fir were included.

The SNP rate reported in the present study (identified by reference-based approaches) is higher than that reported for some woody plants, e.g., 2.6 SNPs per kb in *Populus trichocarpa*^58^. However, comparable SNP frequencies have been reported in *Citrus sinensis* (6 SNPs per kb)^59^, and higher frequencies have been reported in different genes of various woody plants, including four *Eucalyptus* species, i.e., one SNP every 33 bp for *E. nitens*, every 31 bp for *E. globulus*, every 16 bp for *E. camaldulensis*, every 17 bp for *E. loxophleba*^60^, and one SNP every 21.9 bp for *Olea europaea* L^61^.

The ratio of transition to transversion can be used as a quality control measurement, as a significant deviation from expected value could indicate bias and false positives^62^. Even though the expected ratio might vary among species, the identified ratio in this study (1.84) is comparable to the ratios reported in other studies, e.g., 1.66 in pear^63^ and 1.6 in cucumber^64^. Despite the existence of twice as many potential transversion mutations as potential transitions (8 vs. 4), the more frequent occurrence of transition mutations is a common phenomenon. This is because transitions tend to be more conservative in terms of their effect on proteins^65^.

We tested a subset of the identified SNPs by clone analysis on 342 individuals representing 140 clones from the Ambrolauri gene pool (one of the two gene pools initially used for SNP identification). The capacity of the SNPs to find high degree relatedness between individuals was demonstrated by comparing the mismatch profile between ramets and unrelated individuals. The mismatch between a pair of ramets in the present study ranged from 0.5 to 7.77%. Telfer et al.^66^ reported a comparable result in their exome capture genotype-by-sequencing SNP panel for radiata pine. In their study, the mismatch percentage between pairs of ramets in the most effective panel ranged from 0 to 7.87%. Identity analysis and the genomic relatedness matrix further demonstrated the effectiveness of the identified SNPs in relatedness analysis. Moreover, it pinpointed the presence of relatedness among some of the clones, which are otherwise assumed to be unrelated. The Ambrolauri gene pool material used in our study was selected from a stand of around 40,000 Christmas trees produced from a commercial seed lot, out of which 200 of the best-performing individuals were initially selected. Out of these, around 140 trees are still contained in the FP.266 CSO, while the remaining have been thinned away due to early bud flushing (= risk of frost damage), poor post-harvest needle retention, or the bad appearance of shoots/needles. Thus, the presence of high relatedness among some individuals might be explained by the possibility of having unknowingly selected the best-performing trees of the same families to establish the clonal orchard, from which the test individuals were selected. This further demonstrates the potential of the identified SNPs in resolving different types of relatedness. We believe that the identified SNPs will be of great value in the ongoing breeding program of the species. They will enable marker-based pedigree reconstruction or the computation of a genomic relatedness matrix to perform large scale ad hoc breeding in Nordmann fir. In addition, the developed SNPs will serve as an important long-term resource for genetic, ecological, and evolutionary studies of the species. The cost of genotyping and/or targeted sequencing in our study was 7.2 USD per sample. Even though the per-sample cost is not specified, Lin et al.^67^ reported that the cost of genotyping per sample of their amplicon-based targeted sequencing panel was 70% less than the array-based method in *Pinus taeda*.

Since the applied references are from a related species or low copy region assembly from Nordmann fir, the SNP frequency reported in the present study may deviate from the actual level of SNPs in the genome. The polymorphism of some of the SNPs might be limited to the gene pool used to identify them. Thus, individuals from both gene pools used in the identification phase should be used to validate the remaining SNPs to avoid ascertainment bias. Future genomic studies on the species should focus on the development of a high-quality reference genome, ideally a pangenome to capture as much variability as possible.

## Conclusions

We have successfully identified a large pool of SNPs in Nordmann fir by exploiting a combination of approaches. The study demonstrates how a closely related species’ reference genome, combined with low coverage whole genome sequencing, can be used to identify a set of reliable SNPs in a species with a complex mega-genome such as that of Nordmann fir. After employing different filtration criteria, 56.5% of the tested SNPs were retained, suggesting that a large portion of the identified SNPs could be used for downstream applications such as pedigree reconstruction, clone identification, and genomic selection.

### Supplementary Information

Supplementary Information.

## Data Availability

The whole genome sequencing reads generated and analyzed during the current study, as well as the low copy region assembly and identified SNPs, are publicly available from https://erda.ku.dk/archives/750eaa788a2a7e6c3fd4ad727772207a/published-archive.html.
